# Dietary antioxidants and hypertension among menopausal women in Rafsanjan Cohort Study

**DOI:** 10.1038/s41598-024-63401-4

**Published:** 2024-06-03

**Authors:** Marzieh Najar, Parvin Khalili, Fatemeh Ayoobi, Mohadese Rezaei Poor, Hajar Vatankhah, Hadi Pourmirzaei Olyaei, Reza Vazirinejad, Zahra Jamali

**Affiliations:** 1 Department of Midwifery, School of Nursing and Midwifery, Geriatric Care Research Center, Rafsanjan University of Medical Sciences, Rafsanjan, Iran; 2 Department of Epidemiology, School of Public Health, Social Determinants of Health Research Center, Rafsanjan University of Medical Sciences, Rafsanjan, Iran; 3 Occupational Safety and Health Research Center, NICICO, World Safety Organization and Rafsanjan University of Medical Sciences, Rafsanjan, Iran; 4 Clinical Research Development Unit (CRDU), Ali-Ibn Abi-Talib Hospital, Rafsanjan University of Medical Sciences, Rafsanjan, Iran; 5 Department of Obstetrics and Gynecology, School of Medicine, Rafsanjan University of Medical Sciences, Rafsanjan, Iran; 6 Molecular Medicine Research Center, Research Institute of Basic Medical Sciences, Rafsanjan University of Medical Sciences, Rafsanjan, Iran; 7 Department of Nutrition, Science and Research Branch, Islamic Azad University, Tehran, Iran; 8 Pistachio Safety Research Center, Rafsanjan University of Medical Sciences, Rafsanjan, Iran; 9 Non-Communicable Diseases Research Center, Rafsanjan University of Medical Sciences, Rafsanjan, Iran; 10 Clinical Research Development Unit (CRDU), Niknafs Hospital, Rafsanjan University of Medical Sciences, Rafsanjan, Iran

**Keywords:** Antioxidants, Dietary intake, Hypertension, Blood pressure, Menopause, Women, Prospective Epidemiological Research Studies in IrAN (PERSIAN), Rafsanjan Cohort Study (RCS), Medical research, Nutrition disorders

## Abstract

Studies on the beneficial role of dietary antioxidants in preventing or managing hypertension in postmenopausal women are infrequent. The present cross-sectional study aimed to assess the association between dietary antioxidants and hypertension among menopausal women in Rafsanjan, a city located in the southeast of Iran. This study was based on data from the Rafsanjan Cohort Study (RCS), as part of the Prospective Epidemiological Research Studies in IrAN (PERSIAN). Among 2359 postmenopausal women, finally, 1936 women were included in this study. Participants were grouped as having normal blood pressure (BP), elevated BP, stage 1 hypertension, or stage 2 hypertension as defined by the 2017 American College of Cardiology (ACC)/American Heart Association (AHA) BP guideline. A food frequency questionnaire (FFQ), was utilized to ascertain the levels of various nutrients and dietary antioxidants in the diet. The association between dietary intakes of antioxidants and blood pressure groups was evaluated by crude and adjusted models in the multinominal logistics regression analysis. Normal BP, elevated BP, stage 1 hypertension, and stage 2 hypertension were observed in 35.69%, 3.62%, 10.59%, and 50.10% of postmenopausal women respectively. In the adjusted model, in subjects with higher consumption of β-carotene, the odds ratios of elevated BP in the 3rd quartile was about 2 times (OR: 2.04 (1.06–3.93) higher than 1st quartile. Also, in subjects with medium quality of DAQS, the odds ratios of elevated BP and stage 1 blood pressure were about 2 times (OR: 2.09 (1.05–4.17) and 1.69 times (OR: 1.69 (1.09–2.63) higher than subjects with low quality respectively. Furthermore, we did not find any statistically significant association between increased intake of dietary antioxidants and decreased odds of hypertension. After controlling the effects of confounding variables, increased dietary intake of selenium, carotenoids, vitamin A, vitamin C, and vitamin E did not decrease the odds of hypertension in postmenopausal women. Accordingly, it is suggested that this association be further investigated in the follow-up phase of this prospective study.

## Introduction

Hypertension is a common comorbidity associated with a variety of diseases from cardiovascular diseases (CVDs) to neurological problems and endocrine diseases^1^. The overall prevalence of hypertension in Iranian adults based on the 2017 American College of Cardiology (ACC)/American Heart Association (AHA) BP guideline was 48.2%^2^. The pathogenesis of hypertension follows a multifactorial pattern and appears to be under the control of lifestyle, demographic features, environmental determinants, and genetic elements^3,4^. The association of oxidative stress with the development of hypertension has been a topic of interest for researchers^5^.

Compared to men, women of reproductive age show a lower risk of hypertension; however, postmenopausal women present a higher risk of hypertension compared to age-matched male counterparts^6,7^. This elevated risk has been attributed to multiple factors ranging from estrogen and 17-β-estradiol deficiencies to vascular dysfunction^8,9^. The sudden fall in sex hormones in the menopause period leads to not only a perturbation in metabolic processes but also to oxidative disbalance^10^. Estrogen deficiency has been suggested to trigger an imbalance between pro- and anti-oxidative forces in cells^11,12^. Oxidative stress is known to nurture a chronic systemic inflammatory status^13^ and promote atherosclerosis mediated partly via overexpressing angiotensin receptor-1 (AT1) and angiotensin-dependent pathways^14,15^.

The management of hypertension in postmenopausal women requires feasible, safe, and non-invasive approaches to prevent its progression and the occurrence of more vascular damage^16^. It has been suggested that antioxidative nutrients can help manage oxidative stress-induced adverse effects during the menopause period, and antioxidants are gaining more and more attention for managing menopause-associated problems, including hypertension^9^. A study revealed that hypertensive postmenopausal women had significantly lower intake of selenium^17^. Nutritional isoflavones and their metabolites have been reported to augment anti-oxidative estrogen-like effects, preventing hypertension in postmenopausal women^18^. Researchers have already investigated the effects of nutritional supplements with anti-oxidative activity such as grape powder^9^, blueberries^19,20^, soya^21^, vitamin E^22^, curcumin^22^, Omega-3^23^, polyunsaturated fatty acids^24^, and green tea^25^ on estrogen deficiency-induced problems, including hypertension and oxidative stress. In another study, ellagic acid consumption was reported to deliver vascular protective effects similar to that of 17-β-estradiol in ovariectomized hypertensive rats, accompanied by a reduction in the production of superoxide radicals and augmentation of antioxidant enzymes^16^. Overall, plant-based diets have been suggested to protect against CVDs due to anti-oxidative and anti-inflammatory effects^26^.

The primary method utilized to evaluate the potential impact of antioxidant intake on health outcomes involved analyzing individual nutrients^27^. This method focused on the effects of specific antioxidants and overlooked the intricate and cumulative dynamics among antioxidants present in foods^27^. Rivas et al. introduced the Dietary Antioxidant Quality Score (DAQS) to address this limitation. This scoring system aggregates various dietary antioxidants and assigns a quantitative score relative to the FDA's recommended intake, aiming to assess the collective influence of antioxidants on health outcomes^27^. Previous studies showed that dietary antioxidants have been associated with hypertension among women^28,29^, but little attention has been paid to dietary antioxidants among postmenopausal women. We are aware of a prospective control study which showed high dietary total antioxidant capacity (TAC) was associated with a reduced risk of hypertension in French women aged 40–70^28^. Furthermore, a high dietary TAC was associated with a decreased odd of hypertension in Iranian women aged 35–65^29^. To our knowledge, no study examined the association between dietary antioxidants and hypertension among Iranian postmenopausal women. Therefore, we aimed to elucidate the association between dietary antioxidants and hypertension in postmenopausal women in Rafsanjan, Iran.

## Methods

### Study design

This cross-sectional study was conducted using the Rafsanjan Cohort Study (RCS)^30^ data with a total number of 9991 individuals as a part of the prospective epidemiological research studies in IrAN (PERSIAN)^31^. Briefly, RCS is a cohort population-based study that was initiated in August 2015 in Rafsanjan, a city in the southeast of Iran^30^. RCS was designed to recruit a total of 10,000 subjects of both genders aged 35–70 years, living in both urban and suburban areas. This sample size estimated by the PERSIAN Cohort Central Scientific Committee for each PERSIAN cohort site supports adequate statistical power. A population of 10,000 individuals would be large enough to detect for detection of a relative risk of 2 for an exposure, (α level of 0.05, β of 90%, for a given 10% prevalence) with 150 incident cases of the outcome of interest during the follow up phase. A total of 150 incident cases for 15 years of follow up equates to 10 cases/year, which is very much realistic to achieve in RCS^31^.

Participants signed the informed written consent letter. Among this population, 2359 subjects were postmenopausal women. After excluding women with a history of hormone replacement therapy (HRT), women who consumed supplementary vitamins, and women who had incomplete medical questionnaires, finally 1936 women were included in the present study. This study was approved by the Ethics Committee of Rafsanjan University of Medical Sciences (Ethical codes: ID: IR.RUMS.REC.1401.237). The protocol of the study was designed according to the PERSIAN^31^. All methods were performed in accordance with the relevant guidelines and regulations.

### Data collection

All participants completed validated questionnaires containing demographic variables, socioeconomic status, cigarette smoking, opium use, medical history, dietary status, and physical activity by trained interviewers. Anthropometric variables were measured by trained personnel. Socio-economic status (wealth score index: WSI) was calculated using multiple correspondence analysis (MCA) of individuals' economic and social variables. Physical activity was assessed by the metabolic equivalent of task (MET) according to 24-h physical activity.

Blood pressure (BP) was taken twice in each arm, and the average of the second measurement in the right and left arms was used to report blood pressure in millimeters of mercury (mmHg). Based on the 2017 ACC/AHA guidelines, women were categorized as having normal BP (untreated SBP < 120 mmHg and DBP < 80 mmHg); elevated BP (untreated SBP 120–129 mmHg and DBP < 80 mmHg); stage 1 hypertension (untreated SBP 130–139 mmHg or DBP 80–89 mmHg); or stage 2 hypertension (SBP_140 mmHg or more, DBP_90 mmHg or more, or under treatment with antihypertensive agents)^32^.

Menopause was defined as the passage of at least 12 months since the last menstruation, which was completed using the reproductive history questionnaire in women.

### Assessment of dietary intake

Food consumption in grams per day was quantified through a food frequency questionnaire (FFQ). This tool was also utilized to ascertain the levels of various nutrients and dietary antioxidants in the diet. The reliability and validity of the FFQ have been established by prior research conducted in Iran^33^.

The Dietary Antioxidant Quality Score (DAQS)^27^ is determined by comparing the intake of six essential micronutrients in daily diets against the recommended daily intake (RDI). These micronutrients include vitamins A, C, and E, as well as selenium, magnesium, and zinc. In this process, a "0" score is allocated for each nutrient if its intake falls below two-thirds of the RDI, and a "1" score is assigned when intake meets or exceeds two-thirds of the RDI. These individual scores are then aggregated, leading to a DAQS total ranging from 0 to 6, where a higher score signifies a more prosperous presence of dietary antioxidants. In this study, the DAQS is categorized into three tiers based on quality: 1–2 points indicating low quality, 3–4 points for medium quality, and 5–6 points reflecting high quality^27^. The dietary intake data, including the intake of these micronutrients and total energy, were derived from the United States Department of Agriculture (USDA) Food and Nutrient Database for Dietary Studies (FNDDS), incorporating the first 24-h intake data and supplements.

Furthermore, the Dietary Antioxidant Index (DAI)^34^ was calculated for each participant using the Food Frequency Questionnaire (FFQ) data. This index is computed by standardizing the amounts of six antioxidant micronutrients- vitamins A, C, E, selenium, manganese, and zinc. The standardization involves subtracting the overall mean from each nutrient amount and dividing it by the total standard deviation (SD). This method helps assess the relative antioxidant content in the participants' diet^34^.

$$DAI=\sum\limits_{i=1}^{n=6}\left(\frac{Individual \;Intake-Mean}{SD}\right)$$

### Statistical analyses

Quantitative variables were described as the mean ± standard deviation or median (IQR) and categorical variables as the frequency and percentage. Also, baseline characteristics of individuals were compared across different groups using the chi-square test for categorical variables, and the one-way ANOVA test or Kruskal–Wallis test for quantitative variables.

The association between dietary intakes of antioxidants and BP groups was evaluated by crude and adjusted models in multivariable multinominal logistic regression analysis. The adjusted multinomial logistic regression model was used to control confounding factors’ effects from the relationship between dietary intakes of antioxidants and BP groups. Variables that had a p-value less than 0.25 in the univariate analysis were considered confounders (age, education years, body mass index, physical activity level, diabetes, family history of hypertension in first-degree relatives and salt intake). Furthermore, we considered variables that relevant epidemiological studies determined to be confounders even though they had a p-value greater than 0.25 in univariate analysis (WSI and cigarette smoking).

The baseline model was stratified on the status of dietary intake of antioxidants. The adjusted model included confounding variables age (continuous variable), education years (continuous variable), WSI (continuous variable), cigarette smoking (yes/no), body mass index (continuous variable), physical activity level (continuous variable), diabetes (yes/no), family history of hypertension in first-degree relatives (yes/no) and salt intake (continuous variable). Also, we calculated the predicted probabilities of BP groups against dietary antioxidants (continuous variable) using multivariable multinominal logistic regression. All p-values were two-sided and a p-value < 0.05 was considered the significance level.

## Results

Table 1 shows some demographic and selected medical characteristics of study participants by blood pressure groups. In the present study, the mean age of the study participants was 58.16 ± 5.73 years. The participants (n = 1936) were categorized as having normal BP (n = 691), elevated BP (n = 70), stage 1 hypertension (n = 205), and stage 2 hypertension (n = 970). Overall, normal BP, elevated BP, stage 1 hypertension, and stage 2 hypertension were observed in 35.69%, 3.62%, 10.59%, and 50.10% of women respectively. Age, education, physical activity, BMI, diabetes, and family history of hypertension in first-degree relatives had significant relationships with hypertension. While, opium consumption, cigarette smoking, WSI and dietary salt intake were not significant variables for hypertension. Participants with stage 2 hypertension were more likely to have diabetes, a family history of hypertension in first-degree relatives, a lower mean of physical activity, and a higher BMI (Table 1). Some demographic characteristics and dietary antioxidants of study participants by DAQS groups have been shown in supplementary data (Table S1). Participants with low quality of DAQS were more likely to be older and had a lower mean of physical activity, BMI, education and WSI.

**Table 1:** Demographic and selected medical characteristics of study participants by blood pressure groups (n = 1936).

Characteristics	Overall (n = 1936)	Blood pressure groups				
		Normal (n = 691)	Elevated (n = 70)	Stage 1 hypertension (n = 205)	Stage 2 hypertension (n = 970)	p-value^#^
Age (years)						< 0.001
Mean (SD)	58.16 (5.73)	56.65 (5.85)	60.00 (5.06)	56.77 (5.17)	59.40 (5.46)	
Education (years)						0.030
Mean (SD)	4.86 (4.56)	5.11 (4.62)	4.14 (3.95)	5.36 (4.56)	4.62 (4.55)	
Physical activity						< 0.001
Mean (SD)	37.34 (3.41)	37.72 (3.30)	37.71 (3.83)	37.75 (3.62)	36.94 (3.36)	
BMI (kg/m^2^)						< 0.001
Mean (SD)	29.83 (4.88)	28.62 (4.46)	28.82 (4.93)	30.20 (4.97)	30.68 (5.00)	
WSI						0.283
Mean (SD)	− 0.415 (1.043)	− 0.396 (1.082)	− 0.395 (0.944)	− 0.306 (0.955)	− 0.453 (1.039)	
Cigarette smoking—no (%)						0.648
Yes	79 (4.08)	27 (3.91)	1 (1.43)	8 (3.90)	43 (4.44)	
No	1855 (95.92)	664 (96.09)	69 (98.57)	197 (96.10)	925 (95.56)	
Opium consumption—no (%)						0.180
Yes	112 (5.79)	36 (5.21)	8 (11.43)	10 (4.88)	58 (5.99)	
No	1822 (94.21)	655 (94.79)	62 (88.57)	195 (95.12)	910 (94.01)	
Diabetes—no (%)						< 0.001
Yes	715 (36.93)	179 (25.90)	23 (32.86)	59 (28.78)	454 (46.80)	
No	1221 (63.07)	512 (47.10)	47 (67.14)	146 (71.22)	516 (53.20)	
Family history of hypertension in first-degree relatives—no (%)						< 0.001
Yes	1163 (60.07)	333 (48.19)	38 (54.29)	119 (58.05)	673 (69.38)	
No	773 (39.93)	358 (51.81)	32 (45.71)	86 (41.95)	297 (30.62)	
Salt (g/day)						0.193
Mean (SD)	2.53 (1.72)	2.62 (1.60)	2.45 (1.48)	2.64 (1.33)	2.46 (1.89)	

^#^The mean (SD) was obtained from ANOVA test and frequency and percentage were obtained from chi-square test.

SD: standard deviation, WSI: wealth score index.

Table 2 demonstrates the description of dietary antioxidants by the BP groups in the study participants. We did not find any significant association between the mean of dietary antioxidants and the BP groups.

**Table 2:** The description of dietary antioxidants by the blood pressure groups in the study participants (n = 1936).

Characteristics	Blood pressure groups				
	Normal (n = 691)	Elevated (n = 70)	Stage 1 Hypertension (n = 205)	Stage 2 Hypertension (n = 970)	p-value^#^
Se (µg/day)					0.151
Median (IQR)	47.31 (30.14–70.22)	51.18 (36.37–70.04)	49.03 (34.61–77.72)	45.11 (31.07–69.94)	
Β-carotene (µg/day)					0.055
Median (IQR)	2080.25 (1322.88–3272.90)	2688.09 (1425.91–3586.56)	2371.27 (1427.33–3622.54)	2243.80 (1320.05–3454.18)	
α-Carotene (µg/day)					0.389
Median (IQR)	283.31 (131.90–562.67)	302.13 (149.86–714.64)	293.34 (131.46–667.23)	290.98 (147.09–614.14)	
Lycopene (µg/day)					0.242
Median (IQR)	6381.67 (4337.09–9597.62)	6911.16 (3764.18–11,608.66)	7075.00 (4725.83–10,719.15)	6511.55 (4309.36–10,149.37)	
Vit A (IU)					0.072
Median (IQR)	4572.69 (3082.88–6985.40)	5683.77 (3391.38–7689.42)	5079.37 (3327.31–7526.48)	4829.00 (3008.63–7233.13)	
Vit C (mg/day)					0.388
Median (IQR)	73.53 (46.97–114.29)	76.74 (45.23–133.45)	80.13 (52.99–116.26)	75.78 (48.16–116.72)	
Β-cryptoxanthin (µg/day)					0.519
Median (IQR)	137.47 (75.53–242.56)	140.72 (72.64–251.19)	150.10 (93.18–236.50)	141.06 (82.02–232.31)	
Vit E (mg/day)					0.828
Median (IQR)	4.04 (3.01–5.46)	4.17 (3.16–5.52)	4.14 (3.06–5.37)	4.11 (2.99–5.54)	
Magnesium (mg/day)					0.098
Median (IQR)	229.55 (179.48–290.14)	256.14 (192.78–304.59)	236.97 (193.01–309.63)	230.38 (185.97–294.61)	
Zink (mg/day)					0.107
Median (IQR)	6.90 (5.20–8.42)	7.07 (5.90–8.35)	7.07 (5.85–9.00)	6.80 (5.36–8.68)	

^#^The median (IQR) was obtained from Kruskal–Wallis test.

IU: International Units, IQR: Inter quartile range.

Table 3 shows the association between dietary antioxidants and the odds of elevated BP, stage 1 hypertension, and stage 2 hypertension in study participants using the crude and adjusted models. In the fully adjusted model, in subjects with higher consumption of β-carotene, the odds ratios of elevated BP in the 3rd quartile were about 2 times (OR: 2.04 (1.06–3.93) higher than the 1st quartile. Also, in subjects with medium quality of DAQS, the odds ratios of elevated BP were about 2 times (OR: 2.09 (1.05–4.17) higher than subjects with low quality.

**Table 3:** Association of the dietary antioxidants and the odds of elevated blood pressure, stage 1 hypertension, and stage 2 hypertension in study participants using the crude and adjusted models.

Antioxidants	Elevated blood pressure		Stage 1 hypertension		Stage 2 hypertension	
	Crude model	Adjusted model	Crude model	Adjusted model	Crude model	Adjusted model
DAI						
Quartile 1	1	1	1	1	1	1
Quartile 2	1.22 (0.60–2.50)	1.41 (0.68–2.93)	**1.58 (1.01–2.46)**	1.49 (0.95–2.36)	1.11 (0.85–1.45)	1.18 (0.87–1.59)
Quartile 3	1.51 (0.76–3.01)	1.75 (0.85–3.63)	1.32 (0.83–2.10)	1.17 (0.72–1.90)	1.10 (0.83–1.43)	1.05 (0.77–1.43)
Quartile 4	1.31 (0.64–2.67)	1.69 (0.78–3.67)	**1.60 (1.02–2.51)**	1.40 (0.86–2.27)	1.07 (0.81–1.41)	1.10 (0.79–1.52)
DAQS						
1–2 (low-quality)	1	1	1	1	1	1
3–4 (medium-quality)	1.93 (0.99–3.76)	**2.09 (1.05–4.17)**	**1.85 (1.20–2.84)**	**1.69 (1.09–2.63)**	1.21 (0.95–1.55)	1.16 (0.88–1.53)
5–6 (high-quality)	1.44 (0.71–2.92)	1.81 (0.84–3.91)	**1.79 (1.15–2.77)**	1.58 (0.98–2.53)	1.07 (0.83–1.37)	1.04 (0.77–1.40)
Se (µg/day)						
Quartile 1	1	1	1	1	1	1
Quartile 2	1.33 (0.70–2.54)	1.54 (0.79–3.00)	1.05 (0.70–1.59)	1.01 (0.66–1.54)	0.99 (0.77–1.27)	1.08 (0.81–1.42)
Quartile 3	1.46 (0.75–2.85)	1.73 (0.86–3.45)	1.13 (0.73–1.74)	1.07 (0.69–1.67)	0.87 (0.66–1.14)	0.90 (0.67–1.23)
Quartile 4	1.37 (0.67–2.80)	1.47 (0.70–3.07)	1.31 (0.85–2.03)	1.19 (0.76–1.87)	0.97 (0.73–1.28)	0.92 (0.67–1.26)
Β-carotene (µg/day)						
Quartile 1	1	1	1	1	1	1
Quartile 2	0.57 (0.25–1.27)	0.60 (0.26–1.36)	1.03 (0.66–1.59)	0.97 (0.62–1.52)	0.98 (0.75–1.27)	0.97 (0.73–1.30)
Quartile 3	1.82 (0.98–3.39)	**2.04 (1.06–3.93)**	1.43 (0.93–2.19)	1.32 (0.84–2.07)	1.26 (0.96–1.65)	1.25 (0.92–1.69)
Quartile 4	1.31 (0.66–2.59)	1.54 (0.74–3.24)	1.48 (0.96–2.28)	1.31 (0.82–2.10)	1.19 (0.91–1.57)	1.16 (0.84–1.60)
α-Carotene (µg/day)						
Quartile 1	1	1	1	1	1	1
Quartile 2	1.21 (0.60–2.47)	1.37 (0.67–2.81)	0.80 (0.51–1.25)	0.74 (0.47–1.17)	1.18 (0.90–1.55)	1.23 (0.91–1.66)
Quartile 3	0.97 (0.46–2.02)	0.96 (0.45–2.05)	0.93 (0.61–1.42)	0.83 (0.54–1.29)	1.12 (0.85–1.47)	1.00 (0.74–1.35)
Quartile 4	1.70 (0.87–3.31)	1.78 (0.88–3.60)	1.12 (074–1.71)	0.99 (0.64–1.55)	1.26 (0.95–1.65)	1.14 (0.83–1.56)
Lycopene (µg/day)						
Quartile 1	1	1	1	1	1	1
Quartile 2	0.87 (0.44–1.72)	0.95 (0.48–1.92)	1.27 (0.83–1.95)	1.26 (0.82–1.95)	1.00 (0.77–1.30)	1.08 (0.81–1.44)
Quartile 3	0.85 (0.42–1.71)	1.01 (0.49–2.08)	1.18 (0.76–1.83)	1.09 (0.69–1.71)	0.82 (0.63–1.07)	0.84 (0.62–1.13)
Quartile 4	1.47 (0.77–2.82)	1.82 (0.92–3.60)	1.54 (0.99–2.40)	1.44 (0.91–2.29)	1.13 (0.85–1.49)	1.24 (0.91–1.70)
Vit A. IU						
Quartile 1	1	1	1	1	1	1
Quartile 2	0.74 (0.35–1.56)	0.78 (0.36–1.67)	1.11 (0.73–1.71)	1.05 (0.68–1.62)	0.96 (0.74–1.24)	0.91 (0.68–1.22)
Quartile 3	1.60 (0.85–3.01)	1.81 (0.93–3.52)	1.30 (0.85–1.99)	1.17 (0.75–1.83)	1.13 (0.87–1.48)	1.08 (0.80–1.46)
Quartile 4	1.49 (0.76–2.94)	1.75 (0.84–3.64)	**1.59 (1.03–2.45)**	1.44 (0.90–2.30)	1.24 (0.93–1.63)	1.21 (0.88–1.67)
Vit C (mg/day)						
Quartile 1	1	1	1	1	1	1
Quartile 2	0.87 (0.44–1.70)	0.93 (0.46–1.88)	1.27 (0.84–1.93)	1.18 (0.77–1.81)	1.11 (0.86–1.43)	1.13 (0.85–1.51)
Quartile 3	0.75 (0.36–1.57)	0.87 (0.40–1.92)	1.52 (1.00–2.31)	1.35 (0.86–2.11)	1.05 (0.80–1.37)	1.05 (0.77–1.44)
Quartile 4	1.63 (0.87–3.07)	1.88 (0.93–3.81)	1.11 (0.69–1.78)	0.95 (0.57–1.58)	1.18 (0.89–1.56)	1.17(0.84–1.63)
β-Cryptoxanthin (µg/day)						
Quartile 1	1	1	1	1	1	1
Quartile 2	0.65 (0.03–1.30)	0.67 (0.33–1.37)	1.29 (0.85–1.95)	1.17 (0.76–1.80)	1.21 (0.94–1.56)	1.20 (0.90–1.61_
Quartile 3	0.83 (0.42–1.63)	0.81 (0.40–1.68)	1.41 (0.92–2.15)	1.17 (0.74–1.84)	1.05 (0.80–1.37)	0.89 (0.65–1.22)
Quartile 4	1.05 (0.55–2.02)	1.10 (0.53–2.28)	1.04 (0.65–1.65)	0.85 (0.51–1.43)	1.03 (0.78–1.36)	0.98 (0.70–1.37)
Vit E (mg/day)						
Quartile 1	1	1	1	1	1	1
Quartile 2	1.09 (0.60–1.99)	1.17 (0.63–2.20)	1.01 (0.68–1.49)	0.93 (0.62–1.39)	0.95 (0.74–1.21)	0.91 (0.69–1.19)
Quartile 3	0.82 (0.39–1.70)	1.03 (0.48–2.21)	1.16 (0.76–1.76)	1.04(0.67–1.62)	0.97 ((0.74–1.27)	1.02 (0.75–1.38)
Quartile 4	1.26 (0.60–2.65)	1.69 (0.75–3.82)	1.03 (0.62–1.71)	0.84 (0.49–1.45)	1.05 (0.77–1.43)	1.04 (0.73–1.50)

Crude model: the baseline model was not adjusted.

Adjusted model: the adjusted model is for confounding variables including age (continuous variable), education years (continuous variable), WSI (continuous variable), cigarette smoking (yes/no), body mass index (continuous variable), physical activity level (continuous variable), diabetes (yes/no), family history of hypertension in first-degree relatives (yes/no) and salt intake (continuous variable).

Subjects were grouped as having normal BP (untreated SBP < 120 mmHg and DBP < 80 mmHg); elevated BP (untreated SBP 120–129 mmHg and DBP < 80 mmHg); stage 1 hypertension (untreated SBP 130–139 mmHg or DBP 80–89 mmHg); or stage 2 hypertension (SBP ≥ 140 mmHg, DBP ≥ 90 mmHg, or taking antihypertensive drugs).

IU: International Units, DAI: dietary antioxidant index, DAQS, dietary antioxidant quality score.

Significant values are in bold.

In the crude model, the odds ratio of Stage 1 blood pressure in subjects with medium quality of DAQS, was 1.85 times (OR: 1.85 (1.20–2.84) higher than subjects with low quality. This positive association remained significant after adjusting for all confounder variables (OR: 1.69 (1.09–2.63). In the crude model, the odds ratio of Stage 1 blood pressure in subjects with high quality of DAQS, was 1.79 times (ORs: 1.79 (1.15–2.77) higher than in subjects with low quality. However, this positive association was not observed after adjusting for all confounder variables (OR: 1.58 (0.98–2.53). Also, we calculated the predicted probabilities of normal BP, elevated BP, stage 1 hypertension, and stage 2 hypertension and graphed them against dietary antioxidants (continuous variable) using multivariable multinominal logistic regression (Fig. S1).

## Discussion

In the current cross-sectional study with a large sample size of menopausal women (n = 1936), the associations between dietary antioxidant intake of selenium, carotenoids (α-carotene, beta-carotene, lycopene, and beta-cryptoxanthin), vitamin A, vitamin C, and vitamin E and also DAI and DAQS scores and hypertension was investigated. We did not find any statistically significant association between increased intake of mentioned antioxidants and decreased odds of hypertension. We found that in people with higher beta-carotene intake, the odds ratio of elevated blood pressure was higher compared to the reference group. Furthermore, the odds ratios of elevated blood pressure and stage 1 hypertension were higher in people with medium quality of DAQS compared to low quality of DAQS.

The prevalence of elevated BP, stage 1 hypertension, and stage 2 hypertension in this population were 3.62%, 10.59%, and 50.10%, respectively. We found that there was a significant association between increasing age and high blood pressure, which was consistent with some previous studies^35,36^. Inflammation, oxidative stress, and endothelial dysfunction have been suggested as mechanisms of aging-related hypertension^37^. Also, the association between low education level and high blood pressure was significant, as in the study of Veisani et al., the education level was lower in people with high blood pressure^38^. The reason could be a lack of adherence to the recommended lifestyle behaviors to control blood pressure. In terms of BMI, the association between high blood pressure and high BMI was also significant in participants in the second stage of hypertension. Similarly, in the studies of Diaz et al. and Chalwe et al. obesity was related to high blood pressure^35,39^. Fat cells produce high amounts of inflammatory cytokines and adipokines. Adipokines are associated with reduced production and utilization of nitric oxide, which have important functions in controlling vascular tone and suppressing the proliferation of vascular smooth muscle cells. The decreased effect of nitric oxide is associated with endothelial dysfunction and increased arterial blood pressure^40^. In the present study, smoking was not a significant variable for hypertension, but Gać et al. showed that smoking was an independent risk factor for lower total antioxidant status and was associated with greater intima-media thickness leading to hypertension^41^. Perhaps the reason for this difference can be found in the different definitions of a smoker, study design, and different demographic characteristics such as age and sex, and also, different cultures because smoking is not common among women in this region.

In terms of dietary intake of antioxidants and blood pressure groups, we did not find any statistically significant association between increased intake of antioxidants and decreased odds of hypertension. This was inconsistent with some previous studies^28,29^. Villaverde et al. found that high antioxidant capacity was associated with a reduced risk of hypertension in a large group of French women. The reason for this difference may be the cross-sectional nature of our study and their cohort study which reached this conclusion after 12.7 years of follow-up of 40,576 French women. Also, the antioxidant indices investigated were different in these two studies. In the present study, antioxidant quantity and quality indicators (DAI and DAQS) and in the study of Villaverde et al., total antioxidant capacity (dietary TAC) were investigated^28^. In the study of Fateh et al. on 5067 Iranian Kurdish women, a high TAC was associated with a reduced chance of hypertension. After adjusting for confounding variables, participants in the 4th quartile of TAC had a 22% lower chance of hypertension compared to the 1st quartile^29^. Tursunović et al. investigated food habits and nutritional status and their correlation with some health disorders among a sample of 300 postmenopausal women aged 45 to 55 years old. The food questionnaire was based on the Likert scale. Among this sample, the prevalence of hypertension in the period of menopause was 57%. Food habits of women showed insufficient knowledge of nutritional needs and recommendations in this period of life^42^. The association of Indices of diet quality, including dietary diversity score (DDS), Mediterranean dietary score (MDS), diet quality index-international (DQI-I), and healthy eating index-2015 (HEI-2015) with the odds of hypertension among 10,111 individuals (45.14% male) in the Fasa Cohort Study, Iran was investigated. In a fully adjusted model, DQI-I and DDS were not related to the odds of hypertension. While higher adherence to the HEI-2015 (in whole papulation and for both males and females) and MDS (only in whole papulation not in subgroup analysis by gender) was significantly associated with decreased odds of hypertension^43^.

The findings of Kim et al. suggested that antioxidant-rich diets are beneficial in reducing the risk of all-cause mortality and CVD^44^. Wang also concluded that consuming a diet rich in antioxidants significantly prevents cardiovascular mortality^45^. Liu et al. found that vitamins as antioxidants, especially vitamins A, C, and E, can play a role in protecting postmenopausal women from cardiovascular diseases^46^. However, in a clinical trial study conducted by Czernichow et al., no association was found between antioxidant supplementation and blood pressure reduction^47^. This suggests that a natural balance of dietary antioxidants may be more effective in preventing high blood pressure than specific supplements. The use of antioxidant supplements may lead to an excessive number of certain antioxidants, resulting in an imbalance of the complex antioxidant system.

Also, we found that in people with higher beta-carotene intake, the odds ratio of elevated blood pressure was higher compared to reference group after controlling the effects of confounding variables. In contrast to our study, Zhu et al. found that high serum carotenoid concentrations were associated with lower risks of all-cause and cardiovascular mortality in hypertensive adults^48^. The reason for this difference can be the type of study (the cross-sectional nature of our study vs. their cohort study), demographic characteristics (age range, race, gender), different outcome (hypertension vs. all case mortality) and the different measurements of carotenoids (estimated dietary intake by FFQ vs. serum measurement). Furthermore, in the present study, the odds ratios of elevated blood pressure and stage 1 hypertension were higher in people with medium quality of DAQS compared to low quality of DAQS. Due to the cross-sectional nature of the present study, probably people with high blood pressure were on the doctor's advice. To reduce blood pressure, they have had a healthy diet, such as a sufficient intake of vegetables and fruit, which may increase DAQS in subjects with elevated hypertension. Based on this, it is suggested that this relationship be done in the follow-up phase of this prospective study.

This study has strengths and limitations. One of the main strengths of our study was its population-based nature with a large sample size and extensive data collection. This study was the first study that investigated the association between dietary antioxidants and hypertension in Iranian postmenopausal women. The present study used new definitions for blood pressure classification based on the 2017 ACC/AHA guidelines, which can be another strong point. Furthermore, all the data and measurements including blood pressure measurements were collected by trained personnel in one center which could reduce the risk of bias due to different devices and personnel. However, our study had some limitations. The cross-sectional design of this study does not allow any causal inference and the possible role of low dietary antioxidants in high blood pressure in postmenopausal women. Second, FFQ was susceptible to recall bias. The amount of food consumed in the last year may have been underestimated or overestimated. Tirth, biochemical testing that has higher accuracy than dietary antioxidant intake was not available in the present study. The pharmacokinetics of dietary antioxidants, including their absorption, metabolism, elimination, and bioavailability, represent a crucial limitation of our study. Water-soluble antioxidants such as vitamin C are quickly absorbed and excreted, which could limit their therapeutic window, whereas fat-soluble antioxidants like vitamin E and carotenoids accumulate in liver and adipose tissues, influencing their long-term effectiveness. Additionally, the correlation between dietary intake and plasma levels of these antioxidants underscores the complexity of predicting their biological efficacy. Studies indicate a positive relationship that could enhance their protective roles^49–51^, yet individual metabolic and genetic differences lead to unpredictable outcomes. A comparison of the relationships among dietary antioxidants and plasma levels of these antioxidants with hypertension among postmenopausal women may provide a more comprehensive understanding of this topic.

## Conclusion

Increased dietary intake of selenium, carotenoids, vitamin A, vitamin C, and vitamin E did not decrease the odds of hypertension in postmenopausal women. Accordingly, it is suggested that this association be further investigated in the follow-up phase of this prospective study.

### Supplementary Information

Supplementary Figure S1.

Supplementary Table S1.

## Data Availability

The datasets used during the current study are available at the Persian Adult Cohort Study Center, Rafsanjan University of Medical Sciences, Iran. The data is not available publicly. However, upon a reasonable request, the data can be obtained from the corresponding author Zahra Jamali.
