# Supplementary material for: Dietary antioxidants and hypertension among menopausal women in Rafsanjan Cohort Study
Source: Sci Rep. 2024 Jun 3;14:12703. doi: 10.1038/s41598-024-63401-4 (PMC11148154; doi:10.1038/s41598-024-63401-4)
Supplement: Supplementary file 1 — Supplementary Figure S1. [file 41598_2024_63401_MOESM1_ESM.docx]

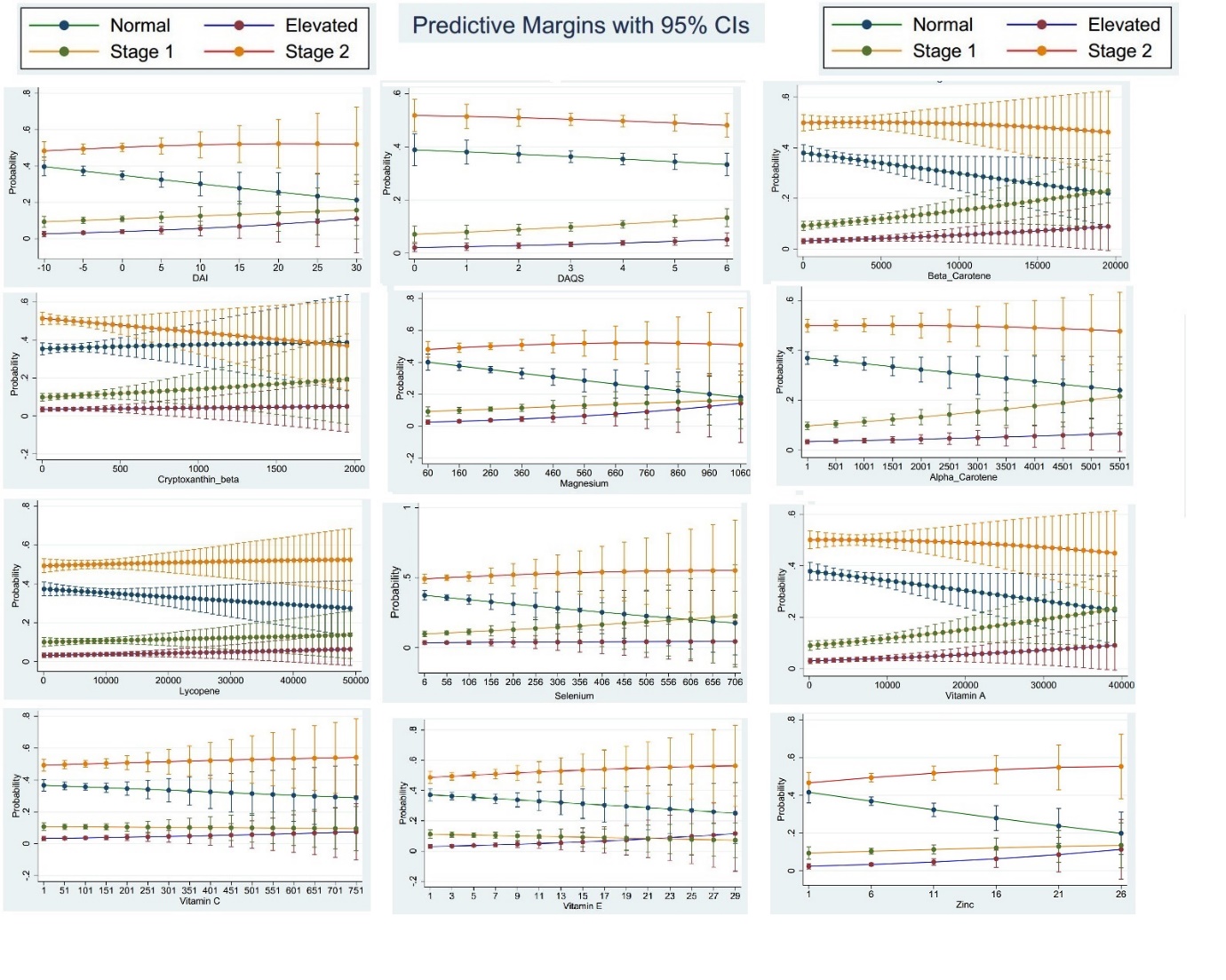


**Figure S1**. Association of the dietary antioxidants (continues variable) and the probability of blood pressure groups.
