# Supplementary material for: Dietary antioxidants and hypertension among menopausal women in Rafsanjan Cohort Study
Source: Sci Rep. 2024 Jun 3;14:12703. doi: 10.1038/s41598-024-63401-4 (PMC11148154; doi:10.1038/s41598-024-63401-4)
Supplement: Supplementary file 2 — Supplementary Table S1. [file 41598_2024_63401_MOESM2_ESM.docx]

| Table S1. Some demographic characteristics and dietary antioxidants of study participants by DAQS groups (n= 1936). | | | | | |
| --- | --- | --- | --- | --- | --- |
| **Characteristics** | **Overall (n=1936)** | **DAQS groups** | | |  |
|  |  | **Low quality (n=691)** | **Medium quality (n=70)** | **High quality (n=205)** | **P-value^#^** |
| **Age. year** | |  |  |  | <0.001 |
| Mean (SD) | 58.15(5.72) | 59.34(5.82) | 58.32(5.46) | 57.07(5.77) |  |
| **Education. year** | |  |  |  | <0.001 |
| Mean (SD) | 4.86(4.56) | 2.92(3.65) | 4.56(4.34) | 6.66(4.75) |  |
| **Physical activity** | |  |  |  | 0.181 |
| Mean (SD) | 37.34(3.40) | 37.12(3.49) | 37.34(3.43) | 37.50(3.31) |  |
| **BMI. kg/m^2^** | |  |  |  | <0.001 |
| Mean (SD) | 29.82(4.88) | 28.82(5.00) | 29.80(4.48) | 30.59(5.12) |  |
| **WSI** | |  |  |  | <0.001 |
| Mean (SD) | -0.415(1.044) | -0.926(1.068) | -0.388(0.95) | -0.071(0.983) |  |
| **Cigarette smoking-no (%)** | |  |  |  | 0.261 |
| Yes | 79(4.09) | 26(5.34) | 30(3.83) | 23(3.48) |  |
| No | 1852(95.91) | 461(94.66) | 754(96.17) | 637(96.52) |  |
| **Opium consumption- no (%)** | | |  |  | 0.908 |
| Yes | 111(5.75) | 28(5.75) | 47(5.99) | 36(5.45) |  |
| No | 1820(94.25) | 459(94.25) | 737(94.01) | 624(94.55) |  |
| **Diabetes- no (%)** | |  |  |  | 0.831 |
| Yes | 714(36.94) | 83(37.58) | 284(36.13) | 247(37.42) |  |
| No | 1219(63.06) | 304(62.42) | 502(63.87) | 413(62.58) |  |
| **Se. µg/day** |  |  |  |  | <0.001 |
| Median (IQR) |  | 25.18(19.63-30.90) | 45.54(35.26-70.73) | 64.55(50.66-92.71) |  |
| **Vit A. IU** |  |  |  |  | <0.001 |
| Median (IQR) |  | 2596.9(1879.10-3479.2) | 4195.33(3311.91-5432.5) | 8022.60(6468.2-10964.16) |  |
| **Vit C.** **mg/day** |  |  |  |  | <0.001 |
| Median (IQR) |  | 38.13(28.41-49.55) | 71.68(54.38-96.74) | 120.95(91.19-157.65) |  |
| **Vit E.** **mg/day** |  |  |  |  | <0.001 |
| Median (IQR) |  | 2.61(1.93-3.30) | 3.90(3.19-4.82) | 5.87(4.70-7.24) |  |
| **Magnesium. mg/day** |  |  |  |  | <0.001 |
| Median (IQR) |  | 159.63(135.29-180.36) | 229.13(202.48-265.16) | 305.27(262.24-364.9) |  |
| **Zink. mg/day** |  |  |  |  | <0.001 |
| Median (IQR) |  | 4.60(3.93-5.16) | 6.80(5.92-7.81) | 8.99(7.77-10.61) |  |
| #The mean (SD) or median (IQR) obtained from ANOVA test and Kruskal-Wallis test respectively and frequency and percentage obtained from chi-square test.  Abbreviations: DAQS: dietary antioxidant quality score, IU: International Units, SD: standard deviation, IQR: Inter quartile range, WSI: wealth score index. | | | | | |
